# Lipidome Profiling of Phosphorus Deficiency-Tolerant Rice Cultivars Reveals Remodeling of Membrane Lipids as a Mechanism of Low P Tolerance

**DOI:** 10.3390/plants12061365

**Published:** 2023-03-18

**Authors:** Soichiro Honda, Yumiko Yamazaki, Takumi Mukada, Weiguo Cheng, Masaru Chuba, Yozo Okazaki, Kazuki Saito, Akira Oikawa, Hayato Maruyama, Jun Wasaki, Tadao Wagatsuma, Keitaro Tawaraya

**Affiliations:** 1 Faculty of Agriculture, Yamagata University, Tsuruoka 997-8555, Japan; 2 Yamagata Integrated Agricultural Research Center, Tsuruoka 997-7601, Japan; 3 RIKEN Center for Sustainable Resource Science, Yokohama 230-0045, Japan; 4 Research Faculty of Agriculture, Hokkaido University, Sapporo 060-8589, Japan; 5 Graduate School of Integrated Sciences for Life, Hiroshima University, Higashi-Hiroshima 739-8521, Japan

**Keywords:** lipidome, phospholipid, phosphorus, P deficiency, remodeling, rice, tolerance

## Abstract

Plants have evolved various mechanisms for low P tolerance, one of which is changing their membrane lipid composition by remodeling phospholipids with non-phospholipids. The objective of this study was to investigate the remodeling of membrane lipids among rice cultivars under P deficiency. Rice (*Oryza sativa* L.) cultivars (Akamai, Kiyonishiki, Akitakomachi, Norin No. 1, Hiyadateine, Koshihikari, and Netaro) were grown in 0 (−P) and 8 (+P) mg P L^−1^ solution cultures. Shoots and roots were collected 5 and 10 days after transplanting (DAT) in solution culture and subjected to lipidome profiling using liquid chromatography-mass spectrometry. Phosphatidylcholine (PC)34, PC36, phosphatidylethanolamine (PE)34, PE36, phosphatidylglycerol (PG)34, phosphatidylinositol (PI)34 were the major phospholipids and digalactosyldiacylglycerol (DGDG)34, DGDG36, 1,2-diacyl-3-O-alpha-glucuronosylglycerol (GlcADG)34, GlcADG36, monogalactosyldiacylglycerol (MGDG)34, MGDG36, sulfoquinovosyldiacylglycerol (SQDG)34 and SQDG36 were the major non-phospholipids. Phospholipids were lower in the plants that were grown under −P conditions than that in the plants that were grown under +P for all cultivars at 5 and 10 DAT. The levels of non-phospholipids were higher in −P plants than that in +P plants of all cultivars at 5 and 10 DAT. Decomposition of phospholipids in roots at 5 DAT correlated with low P tolerance. These results suggest that rice cultivars remodel membrane lipids under P deficiency, and the ability of remodeling partly contributes to low P tolerance.

## 1. Introduction

Phosphorus (P) is an essential nutrient for plant growth and is frequently the major limiting nutrient in soils due to its low availability. P deficiency elicits morphological and physiological changes in the root system and decreases the growth of the plant [1]. Farmers address the issue of P deficiency in soil P by the application of P fertilizer, which is produced from phosphate rock. P is a non-renewable source, and “peak-phosphorus” is expected to happen as early as around 2030 [2]. Phosphorus availability also regulates soil microbial effects on plant performance [3]. Plants have evolved several mechanisms for low P tolerance to cope with low P availability in soil, including (1) higher P acquisition efficiency (PAE) and (2) higher P utilization efficiency (PUE, Wang et al. [4]); however, improvements in PAE and PUE of crop plants are needed to secure food production.

The main mechanisms related to PAE are the expression of high-affinity P transporters, alterations of root system architecture, secretion of phosphatase and organic acids, and association with soil microbes such as mycorrhizal fungi. The main mechanisms relating to PUE include lower P concentration, optimal P distribution, internal P remobilization, lipid remodeling, and alternative P metabolic pathways. 

*Arabidopsis thaliana* copes with P deficiency by replacing phospholipids with nonphosphorous galactolipids [5]. Oats that are grown under P-deficient conditions decrease phospholipid production and increase digalactosyldiacylglycerol (DGDG) production [6]. P-deficient plants use their phospholipids as a major source of internal P supply by replacing phospholipids in their membranes with the non-phosphorus galactolipids [7]. 

Lipidome profiling is the identification and quantification of all lipids in a biological material. Liquid chromatography-mass spectrometry is a useful analytical method for the separation and detection of a wide variety of plant lipids [8]. Lipid remodeling is one of the mechanisms underlying PUE in plants. The ability of lipid remodeling can differ among plant genotypes with different low P tolerances. Reuse of P from P-containing metabolites is an adaptive strategy for plants. Rice is an important crop and staple food for more than half of the world’s population and globally grown on 161 million hectares, with an average annual production of 678.7 million tons [9]. Low P availability in soil is one of the main constraints in rice production [10]. Morphological changes and physiological changes contribute to the P deficiency tolerance of rice [11,12]. However, the relationship between the remodeling ability and the low P tolerance in rice is unknown. The objectives of this study were to identify metabolic alterations in phospholipids and non-phospholipids of rice cultivars under P deficiency with lipidome profiling and to clarify the differences in the lipid replacement ability of among the rice cultivars.

## 2. Results

### 2.1. Growth of 42 Rice Cultivars in Soil Culture

Shoot dry weight of 34 cultivars out of the 42 cultivars was lower in the −P than in the +P (Table S1). Shoot dry weights of eight cultivars showed no difference between −P and +P. The shoot P concentration of 38 cultivars was lower at −P than at +P. The shoot P content of 40 cultivars was lower at −P than at +P. Low P tolerance was different among cultivars and ranged from 33% in Netaro to 76% in Akamai (Figure S1). Akamai, Hiyadateine, and Kiyonishiki were selected as the low P-tolerant cultivars due to the highest value of low P tolerance in the present study. Netaro, Koshihikari, and Norin No.1 were selected as the low P-sensitive cultivars due to the lowest value of low P tolerance in the present study and Akitakomachi were selected as the non-tolerant and non-sensitive cultivars due to the medium value of low P tolerance.

### 2.2. Shoot P Concentration and Shoot Dry Weight of Rice

The shoot P concentrations of all cultivars were lower in the −P plants than in the +P plants at 5 DAT and 10 DAT (Table 1). The shoot P concentration of each P treatment decreased from 5 DAT to 10 DAT, and the degree of decrease was higher in the −P treatment than in the +P treatment. The shoot P concentration of cultivars, except Kiyonishiki, was 1 mg P g^−1^ or less. The shoot dry weights of Akamai, Norin No. 1, and Koshihikari were lower in the −P plants than in the +P plants (Table 1). The shoot dry weight of cultivars except Kiyonishiki were lower in the −P plants than in the +P plants.

### 2.3. Lipid Profiles in Shoots of Seven Rice Cultivars

**Figure 1:**
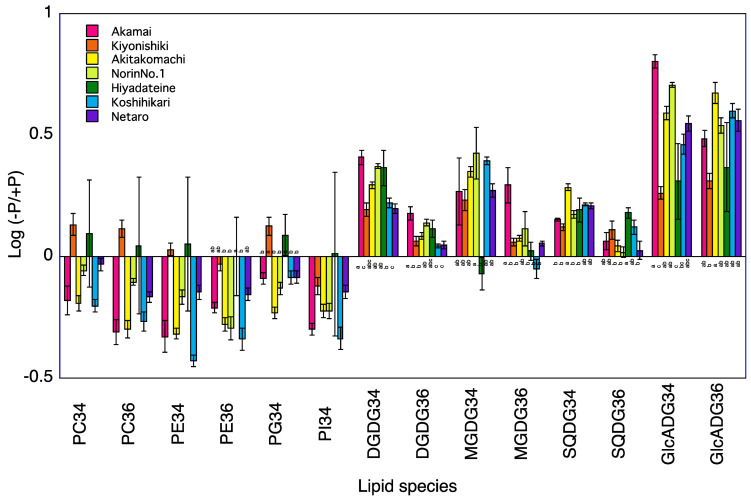
Log (−P/+P) of each lipid species in the shoots of seven rice cultivars 5 days after transplanting. For each lipid species, different letters indicate significant (*p* < 0.05) differences among the seven cultivars.

**Figure 2:**
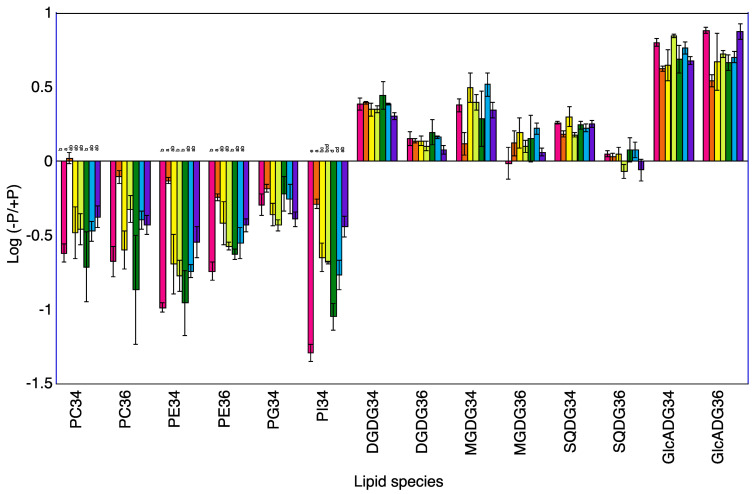
Log (−P/+P) of each lipid species in the shoots of seven rice cultivars 10 days after transplanting. For each lipid species, different letters indicate significant (*p* < 0.05) differences among the seven cultivars.

A total of 120 lipid species were identified in the shoots of seven rice cultivars at 5 and 10 DAT (Table S2). Phosphatidylcholine (PC)34, PC36, phosphatidylethanolamine (PE)34, PE36, phosphatidylglycerol (PG)34, and phosphatidylinositol (PI)34 were the major phospholipids and digalactosyldiacylglycerol (DGDG)34, DGDG36, 1,2-diacyl-3-O-alpha-glucuronosylglycerol (GlcADG)34, GlcADG36, monogalactosyldiacylglycerol (MGDG)34, MGDG36, sulfoquinovosyldiacylglycerol (SQDG)34, and SQDG36 were the major non-phospholipids. The levels of phosphatidylcholine (PC)34, PC36, phosphatidylethanolamine (PE)36, and phosphatidylinositol (PI)34 at 5 and 10 DAT and of phosphatidylglycerol (PG)34 at 10 DAT were lower in the −P plants than in the +P plants for the shoots of Koshihikari (Table 2, Figure 1 and Figure 2). The levels of PE36 and PI34 at 5 and 10 DAT and of PC34, PC36, PE34, and PG34 at 5 DAT were lower in the −P plants than in the +P plants for the shoot of Norin No.1. The levels of PC34, PC36, PE34, PE36, and PI34 at 5 and 10 DAT and of PG34 at 10 DAT were lower in the −P plants than in the +P plants for the shoots of Akamai. The levels of PI34 at 5 and 10 DAT and of PC34, PC36, PE34 and PE36 at 10 DAT were lower in the −P plants than in the +P plants of shoot of Hiyadateine. Levels of PC36, PE34, PE36, and PI34 at 5 and 10 DAT and of PC34 and PG34 at 10 DAT were lower in the −P plants than in the +P plants of shoot of Netaro. The levels of PC34, PC36, PE34, PE36, PG34, and PI34 were lower in the −P plants than in the +P plants of shoot of Akitakomachi. The levels of PC36 and PI34 at 5 and 10 DAT and of PC34 and PG34 at 10 DAT were lower in the −P plants than in the +P plants of shoot of Kiyonishiki.

The levels of digalactosyldiacylglycerol (DGDG)34, 1,2-diacyl-3-O-alpha-glucuronosylglycerol (GlcADG)34, GlcADG36, monogalactosyldiacylglycerol (MGDG)34, and sulfoquinovosyldiacylglycerol (SQDG)34 at 5 and 10 DAT and of DGDG36 and MGDG36 at 10 DAT were higher in the −P plants than in the +P plants for the shoots of Koshihikari (Table 2). The levels of DGDG34, GlcADG34, GlcADG36, and MGDG34 at 5 and 10 DAT and of DGDG36 and SQDG36 at 5 DAT and of SQDG34 at 10 DAT were higher in the −P plants than in +P plants for the shoots of Norin No.1. The levels of DGDG34, DGDG36, GlcADG34, GlcADG36, MGDG34, and SQDG34 at 5 and 10 DAT were higher in the −P plants than in the +P plants for the shoot of Akamai. The levels of DGDG34, DGDG36, GlcADG34, GlcADG36, SQDG34, and SQDG36 at 5 and 10 DAT and of MGDG34 and MGDG36 at 10 DAT were higher in the −P plants than in the +P plants for the shoots of Hiyadateine. The levels of DGDG34, DGDG36, GlcADG34, GlcADG36, MGDG34, MGDG36, and SQDG34 at 5 and10 DAT were higher in the −P plants than in the +P plants for the shoots of Netaro. The levels of DGDG34, DGDG36, GlcADG34, GlcADG36, MGDG34, MGDG36, and SQDG34 at 5 and 10 DAT were higher in the −P plants than in the +P plants for the shoots of Akitakomachi. The levels of DGDG34, DGDG36, GlcADG34, GlcADG36, and SQDG34 at 5 and 10 DAT and of MGDG34 and MGDG36 at 5 DAT were higher in the −P plants than in +P plants for the shoots of Kiyonishiki.

### 2.4. Lipid Profiles in Roots of Seven Rice Cultivars

**Figure 3:**
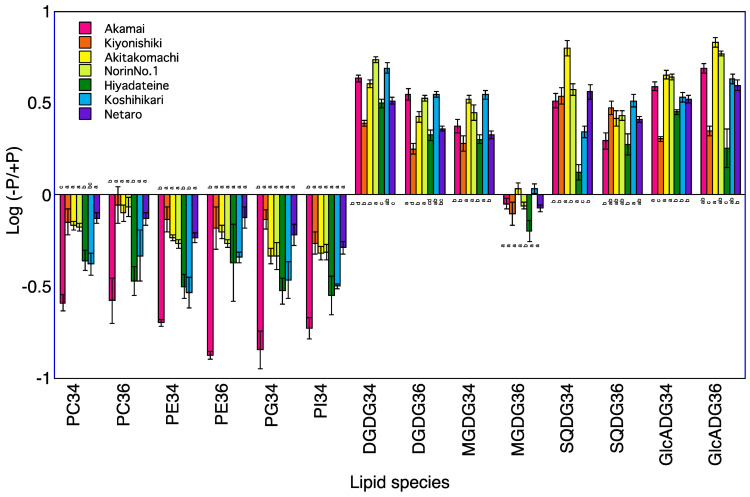
Log (−P/+P) of each lipid species in the roots of seven rice cultivars 5 days after transplanting. For each lipid species, different letters indicate significant (*p* < 0.05) differences among the seven cultivars.

**Figure 4:**
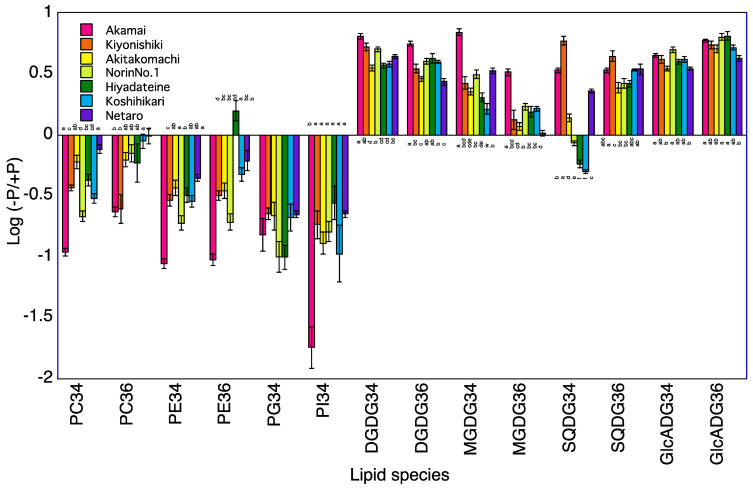
Log (−P/+P) of each lipid species in the roots of seven rice cultivars 10 days after transplanting. For each lipid species, different letters indicate significant (*p* < 0.05) differences among the seven cultivars.

A total of 120 lipid species were identified in the roots of seven rice cultivars at 5 and 10 DAT (Table S3). The levels of PC34, PE34, and PI34 at 5 and 10 DAT and of PG34 at 5 DAT were lower in the −P plants than in +P plants for the roots of Koshihikari (Table 3, Figure 3 and Figure 4). The levels of PC34, PE34, PE36, PG34, and PI34 at 5 and 10 DAT were lower in the −P plants than in the +P plants for the roots of Norin No.1. The levels of PC34, PC36, PE34, PE36, and PI34 at 5 and 10 DAT and of PG34 at 5 DAT were lower in the −P plant than in the +P plants for the roots of Akamai. Levels of PC36 at 5 and 10 DAT and of PC34, PE34, and PG34 at 5 DAT were lower in the −P plants than in the +P plants for the roots of Hiyadateine. The levels of PE34 and PI34 at 5 and 10 DAT and of PC34 and PE34 at 5 DAT and PG34 at 10 DAT were lower in the −P plants than in +P plants for the roots of Netaro. The levels of PC34, PE34, PE36, PG34, and PI34 at 5 and 10 DAT were lower in the −P plants than in the +P plants for the roots of Akitakomachi. The levels of PC34, PC36, PE34, PE36, PG34, and PI34 at 10 DAT were lower in the −P plants than in the +P plants for the roots of Kiyonishiki.

The levels of DGDG34, DGDG36, GlcADG34, GlcADG36, MGDG34, SQDG34, and SQDG36 at 5 and 10 DAT and of MGDG36 at 10 DAT were higher in the −P plants than in the +P plants for the roots of Koshihikari (Table 3). The levels of DGDG34, DGDG36, GlcADG34, GlcADG36, MGDG34, and SQDG36 at 5 and 10 DAT and of SQDG34 at 5 DAT and of MGDG36 at 10 DAT were higher in the −P plants than in the +P plants for the roots of Norin No.1. The levels of DGDG34, DGDG36, GlcADG34, GlcADG36, MGDG34, SQDG34, and SQDG36 at 5 and 10 DAT and of MGDG36 at 10 DAT were higher in the −P plants than in the +P plants for the roots of Akamai. The levels of DGDG34, DGDG36, GlcADG34, MGDG34, MGDG36, and SQDG34 at 5 and 10 DAT and of SQDG36 at 5 DAT and of GlcADG36 at 10 DAT were higher in the −P plants than in the +P plants for the roots of Hiyadateine. The levels of DGDG34, DGDG36, GlcADG34, GlcADG36, MGDG34, SQDG34, and SQDG36 at 5 and 10 DAT were higher in the −P plants than in the +P plants for the roots of Netaro. The levels of DGDG34, DGDG36, GlcADG34, GlcADG36, MGDG34, and SQDG36 at 5 and 10 DAT and of SQDG34 at 5 DAT were higher in the −P plants than in the +P plants for the roots of Akitakomachi. The levels of DGDG34, DGDG36, GlcADG34, GlcADG36, MGDG34, SQDG34, and SQDG36 at 5 and 10 DAT were higher in the −P plants than in the +P plants for the roots of Kiyonishiki.

## 3. Discussion

### 3.1. P Deficiency Increases Phospholipids Decomposition

The degradation of phospholipids for lipid remodeling under P deprivation has been reported in *Avena sativa* [6], *Arabidopsis thaliana* [13], *Emiliania huxleyi* [14], microalgae [15], *Phaseolus vulgaris* [16], and Proteaceae [17,18]. These experiments were carried out using one cultivar or genotype. We clarified that the low P-tolerant rice cultivar Akamai catabolizes more phosphatidylcholine, phosphatidylethanolamine, and phosphatidylgylcerol in older leaves than the low P-sensitive cultivar Koshihikari and synthesized digalactosyldiacylglycerol and monogalactosyldiacylglycerol in younger leaves [19]. However, it is not known whether this difference also occurs among different low P-tolerant cultivars. A total of seven cultivars decreased phospholipids, PI, PG, PE, and PC in the shoots and roots under P deficiency at 5 and 10 DAT (Figure 1, Figure 2, Figure 3 and Figure 4). The degree of decrease in most phospholipids in roots at 5 and 10 DAT was higher than that in the shoots at 5 and 10 DAT. Degradation of PE and PC in mature leaves of *Hakea prostrata* under P deficiency was higher than that in young leaves and the phosphocholine/phosphoethanolamine phosphatase gene expression in mature leaves was higher than that in young leaves [18]. Phosphatidylcholine-hydrolyzing phospholipase C of *A. thaliana* showed transcriptional activation upon P limitation [13]. Higher degradation of PE and PC degradation in rice roots may be related to the activities of these enzymes. The degree of decrease in the PI, PG, PE, and PC content in the shoots and roots at 10 DAT was higher than that at 5 DAT. The difference in the shoot P concentration between −P and +P was higher at 10 DAT than that at 5 DAT. Severe P deficiency at 10 DAT exacerbates the degradation of PI, PG, PE, and PC.

### 3.2. P Deficiency Increases Non-Phospholipid Synthesis

Accumulation of non-phospholipids for lipid remodeling under P deprivation has been observed in *Avena sativa* [6], *Emiliania huxleyi* [14], microalgae [15], *Phaseolus vulgaris* [16], and Proteaceae [17,18]. These experiments were also conducted with one cultivar or genotype. We clarified that the low P-tolerant rice cultivar Akamai synthesizes more non-phospholipids than the low P-sensitive cultivar Koshihikari [19]. It is also not known whether this difference occurs among different low P-tolerant cultivars. A total of seven cultivars increased non-phospholipids, GlcADG, SQDG, MGDG, and DGDG in shoots and roots under P deficiency at 5 and 10 DAT. The degrees of increase in most non-phospholipids in roots at 5 and 10 DAT was higher than those in shoots at 5 and 10 DAT. P-depleted conditions increased mol% of DGDG and SQDG in *Seamum indicum* and up-regulated MGDG synthase gene (*SeMGD1* and *SeMGD2*) [20]. Upregulation of the *SQDG synthase* transcripts level in tomatoes and soybeans was observed [21]. The synthesis of non-phospholipid DGDG [22] and GlcADG [8] in *Arabidopsis* was also reported. MGDG, DGDG, and SQDG synthases may be related to the accumulation of non-phospholipids in rice. The degrees of increase in most non-phospholipids in shoots and roots at 10 DAT was higher than those at 5 DAT. Accumulation of DGDG in 4-week old shoots and roots of *A. sativa* was higher than that in 2-week old plants [6]. The difference in the shoot P concentration between −P and +P was higher at 10 DAT than at 5 DAT. Severe P deficiency at 10 DAT exacerbates the synthesis of GlcADG, SQDG, MGDG, and DGDG.

### 3.3. Relationship between Lipid Remodeling and Low P Tolerance

**Figure 5:**
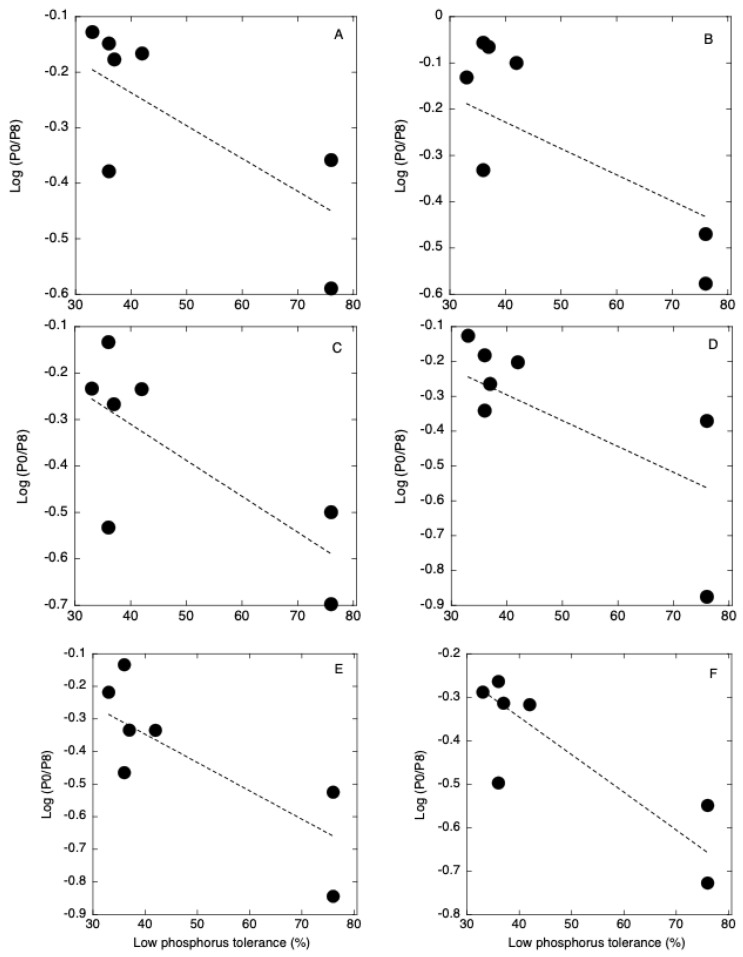
Relationship between low phosphorus tolerance and log (−P/+P) of PC34 (**A**), PC36 (**B**), PE34 (**C**), PE36 (**D**), PG34 (**E**), and PI34 (**F**) in roots at 5 days after transplanting.

The degree of phospholipid decomposition in roots at 5 DAT was negatively correlated with the low P tolerance of seven rice cultivars (Figure 5, Table S4). Low P-tolerant cultivars decomposed more phospholipids than low P-sensitive cultivars. The degrees of decrease in PI, PG, PE, and PC were higher in the roots of Akamai at 5 DAT and degrees of decrease in PI, PE, and PC were higher in the shoots and roots of Akamai at 5 and 10 DAT. The ability to degrade phospholipids in Akamai may contribute to the low P tolerance of this cultivar. Orthophosphate that is produced by decomposition can be used for the synthesis of P-containing compounds such as sugar phosphate, ATP, and nucleic acids. The degrees of increase in GlcADG, SQDG, MGDG, and DGDG were similar among the seven cultivars. The degree of increase in MGDG was higher in the roots of Akamai at 10 DAT. There was no correlation between low P tolerance and the degree of phospholipid decomposition in shoots. Maintenance of membrane lipids in the roots by the remodeling is more important than that in the shoots at this growth stage when root membranes support nutrient uptake. There was no correlation between low P tolerance and degree of non-phospholipid synthesis. Verma et al. (2021) found that there was no correlation between galactolipid synthesis and physiological P use efficiency of rice genotypes [23]. Contribution of non-phospholipid synthesis to the P use efficiency may be different among the seven rice cultivars that were used in this study.

## 4. Materials and Methods

### 4.1. Screening of P-Tolerant and P-Sensitive Rice Cultivars Grown in Soil Culture

We grew 42 Japonica rice (*Oryza sativa* L.) cultivars in soil applied with 4.8 g P kg^−1^, (+P) or without (−P) phosphate fertilizer in a growth chamber (16 h light, 365 µmol m^−2^,s^−1^, at 27 °C; 8 h dark at 27 °C) for 27 days after transplanting (DAT). Subsequently, the shoot P concentration and shoot dry weight analyses were carried out on the samples that were collected 5 and 10 DAT. The low P tolerance value of cultivars was calculated by the ratio of shoot dry weight at −P/shoot dry weight at +P.

### 4.2. Lipidome Profiling for Seven Rice Cultivars Grown in Solution Culture

Rice cultivars Akamai, Kiyonishiki, Akitakomachi, NorinNo.1, Hiyadateine, Koshihikari, and Netaro were used. The seeds were put on sterilized perlite and covered with sterilized perlite, applied with deionized water, and kept in a growth chamber (Biotron LH-350S, NK System, Tokyo) at 27 °C (16 h light 150 µMm^−2^ s^−1^/8 h dark) for seven days. Subsequently, 10 seven-day-old seedlings were transplanted to a paper cup (90 × 140 mm) filled with 545 mL of nutrient solution. The nutrient solution contained the following essential nutrients (mg L^−1^) as described by Wagatsuma et al. (1988): 40 N (NH_4_NO_3_), 20 N (NaNO_3_), 60 K (K_2_SO_4_), 80 Ca (CaCl_2_), 40 Mg (MgSO_4_), 2 Fe (FeSO_4_), 1 Mn (MnSO_4_), 0.01 Cu (CuSO_4_), 0.005 Mo ((NH_4_) _6_Mo_7_O_24_), 0.4 B (H_3_BO_3_), and 0.2 Zn (ZnCl_2_). P concentrations of the nutrient solutions were adjusted to 0 (−P) and 8 (+P) mg P L^−1^ with NaH_2_PO_4_. The pH of the solutions was adjusted daily to 5.0, using 0.1 M NaOH and 0.1 M H_2_SO_4_. Air was continuously supplied to the solutions with vinyl chloride tubes that were connected to an air pump and were replaced every two days. Each P treatment had five replications. Rice plants were grown in a growth chamber at 27 °C (16 h light 150 µMm^−2^s^−1^/8 h dark) for five and ten days.

The shoots and roots were harvested 5 DAT and 10 DAT. The whole plants were washed with tap water and deionized water. Subsamples of the shoots and the roots were separated, and fresh weights were measured. The subsamples were frozen right away at –20 °C and dried at 70 °C for three days, respectively. The frozen shoots and roots were used for lipid concentration measurement. The dried shoots were used to determine the dry weight and P concentration. The dry weights of the shoot subsamples were then measured. Ground shoot subsamples were digested using a HNO_3_-HClO_4_-H_2_SO_4_ (5:2:1) solution. The P concentration in the digested solution was determined calorimetrically using the vanadomolybdate-yellow assay.

### 4.3. Lipidomic Analysis

Crude lipid extracts were prepared and analyzed on a Wasters UPLC Xevo G2 Qtof MS in the positive ion mode as previously reported [21,24].

### 4.4. Statistical Analyses

The data were statistically analyzed using analysis of variance using the statistical software KaleidaGraph 5.0 (Synergy Software, Reading, PA, USA). Comparison of means performed using the least significant difference method at the 5% probability level where the F-value was significant.

## 5. Conclusions

The seven rice cultivars decomposed phospholipids PI, PG, PE, and PC and synthesized non-phospholipids GlcADG, SQDG, MGDG, and DGDG in shoots and roots under P deficiency. Degrees of decomposition of PI, PG, PE, and PC in the shoots and roots and synthesis of GlcADG, SQDG, MGDG, and DGDG in shoots and roots were different among seven rice cultivars. The degree of phospholipid decomposition in the roots was negatively correlated with the low P tolerance of seven rice cultivars. The ability of phospholipid decomposition contributes to the P use efficiency as the component of low P tolerance mechanisms of rice.

## Figures and Tables

**Table 1:** Shoot P concentration and shoot dry weight of rice cultivars that were grown in −P and +P. For each cultivar, different lowercase letters indicate significant (*p* < 0.05) differences between −P and +P treatment.

Cultivars	Treatment	Shoot P Concentration		Shoot Dry Weight		
		(mg P g^−1^)		(mg plant^−1^)		
		5 DAT	10 DAT	5 DAT	10 DAT	
Akamai	−P	2.54 ± 0.10 a	0.98 ± 0.04 a	136.0 ± 3.0 a	363.0 ± 11.0 a	
	+P	9.90 ± 0.11 b	7.14 ± 0.37 b	149.0 ± 4.0 b	706.0 ± 1.0 b	
Kiyonishiki	−P	3.63 ± 0.44 a	2.19 ± 0.16 a	7.7 ± 1.0 a	21.5 ± 1.0 a	
	+P	9.75 ± 0.66 b	9.07 ± 0.31 b	8.4 ± 0.0 a	22.4 ± 2.0 a	
Akitakomachi	−P	2.97 ± 0.11 a	0.97 ± 0.03 a	7.1 ± 1.0 a	17.3 ± 0.0 a	
	+P	10.22 ± 0.27 b	6.96 ± 0.36 b	7.6 ± 0.0 a	30.4 ± 5.0 b	
Norin No.1	−P	2.53 ± 0.05 a	0.86 ± 0.01 a	95.0 ± 3.0 a	180.0 ± 3.0 a	
	+P	9.27 ± 0.69 b	6.64 ± 0.56 b	108.0 ± 3.0 b	342.0 ± 26.0 b	
Hiyadateine	−P	3.19 ± 0.20 a	1.05 ± 0.02 a	8.9 ± 0.0 a	22.0 ± 1.0 a	
	+P	11.07 ± 0.34 b	8.05 ± 0.12 b	9.6 ± 1.0 a	34.5 ± 1.0 b	
Koshihikari	−P	2.72 ± 0.07 a	1.01 ± 0.12 a	72.0 ± 6.0 a	147.0 ± 21.0 a	
	+P	8.67 ± 0.44 b	6.26 ± 0.43 b	74.0 ± 3.0 b	263.0 ± 37.0 b	
Netaro	−P	3.49 ± 0.15 a	1.47 ± 0.03 a	7.4 ± 0.0 a	17.2 ± 1.0 a	
	+P	10.40 ± 0.21 b	6.90 ± 0.20 b	8.4 ± 0.0 a	29.4 ± 1.0 b	

**Table 2:** Non-phospholipid species and phospholipid species that were detected in the shoots of seven cultivars under −P and +P treatment 5 and 10 days after transplanting (DAT). For each cultivar, different letters indicate significant (*p* < 0.05) differences between −P and +P treatment.

Lipid Species	Koshihikari								Norin No.1								Akamai								Hiyadateine								Netaro								Akitakomachi								Kiyonishiki							
	5 DAT				10 DAT				5 DAT				10 DAT				5 DAT				10 DAT				5 DAT				10 DAT				5 DAT				10 DAT				5 DAT				10 DAT				5 DAT				10 DAT			
	−P		+P		−P		+P		−P		+P		−P		+P		−P		+P		−P		+P		−P		+P						−P		+P		−P		+P		−P		+P		−P		+P		−P		+P		−P		+P	
DGDG_34	5.3616	a	3.1267	b	10.9152	a	4.4578	b	7.8770	a	3.2909	b	11.4592	a	4.9968	b	8.0599	a	3.1104	b	9.8775	a	3.9650	b	4.7858	a	2.3152	b	8.3167	a	2.5239	b	7.9142	a	5.0111	b	9.7454	a	4.8061	b	9.1287	a	4.6217	b	11.0602	a	4.6388	b	8.4620	a	5.0525	b	13.2061	a	7.5372	b
DGDG_36	7.6882	a	6.9466	a	14.3118	a	9.8575	b	11.5013	a	8.3420	b	15.2931	a	11.9604	a	12.4606	a	8.2258	b	14.1992	a	9.7847	b	8.8747	a	6.3478	b	12.2270	a	6.8134	b	13.5905	a	12.1261	b	14.7189	a	12.2135	b	13.9443	a	11.4374	b	15.1426	a	10.9655	b	13.1541	a	11.3376	b	11.9844	a	8.6851	b
GlcADG_343	0.4106	a	0.1195	b	0.6119	a	0.1458	b	0.5564	a	0.1271	b	0.6772	a	0.1357	b	0.5613	a	0.1433	b	0.8470	a	0.1890	b	0.4532	a	0.1655	b	1.1200	a	0.1967	b	0.6546	a	0.1830	b	1.0884	a	0.2266	b	0.8383	a	0.2113	b	1.3073	a	0.2438	b	0.4814	a	0.2651	b	0.9776	a	0.2250	b
GlcADG_36	0.3994	a	0.0996	b	0.4446	a	0.0875	b	0.3504	a	0.1004	b	0.4431	a	0.0833	b	0.3832	a	0.1239	b	0.8106	a	0.1058	b	0.2251	a	0.0682	b	0.5165	a	0.0735	b	0.1581	a	0.0352	b	0.3907	a	0.0363	b	0.2387	a	0.0318	b	0.5218	a	0.0600	b	0.1269	a	0.0619	b	0.4416	a	0.1094	b
MGDG_34	0.7984	a	0.3207	b	1.8279	a	0.5317	b	0.9193	a	0.3167	b	2.6777	a	1.0501	b	0.8749	a	0.4065	b	0.8236	a	0.3392	b	0.5259	a	0.5583	a	1.5062	a	0.5691	b	3.6475	a	1.9410	b	4.2732	a	1.8870	b	4.1173	a	1.8295	b	3.7471	a	1.4233	b	2.3695	a	1.3675	b	0.5551	a	0.4065	a
MGDG_36	4.8094	a	5.3192	a	17.1815	a	10.2331	b	9.9968	a	7.4017	a	20.7257	a	16.2440	a	12.0847	a	5.8848	a	11.0500	a	10.5372	a	7.5537	a	6.7931	a	14.7230	a	8.1712	b	26.4800	a	23.3615	b	26.5992	a	22.9215	b	26.6611	a	22.3083	b	25.4037	a	19.6906	b	23.9469	a	20.8229	b	9.2537	a	6.6118	a
PC_34	0.5774	b	0.9145	a	0.4843	b	1.3865	a	0.7501	a	0.8520	a	0.7709	b	2.0325	b	0.8634	b	1.2749	a	0.3161	b	1.2872	a	0.4471	a	0.4321	a	0.1639	b	0.5655	a	6.0609	a	6.4913	a	3.5015	b	7.9569	a	5.4276	b	8.3458	a	1.0718	b	4.1896	a	4.0372	a	2.9329	a	1.0390	b	0.9798	a
PC_36	0.2180	b	0.3976	a	0.2160	b	0.5257	a	0.3044	a	0.3845	a	0.4035	b	0.8042	b	0.3718	b	0.7397	a	0.1287	b	0.5664	a	0.1030	a	0.1137	a	0.0387	b	0.1105	a	1.5022	b	2.2044	a	0.9587	b	2.4878	a	1.5039	b	2.9702	a	0.2502	b	1.2075	a	1.3627	b	1.0164	a	0.2420	b	0.3029	a
PE_34	0.1676	b	0.4464	a	0.1009	b	0.5477	a	0.2082	a	0.3027	a	0.1196	b	0.6479	b	0.2310	b	0.4773	a	0.0513	b	0.4949	a	0.1750	a	0.2363	a	0.0422	b	0.2685	a	1.2859	b	1.7725	a	0.6035	b	1.8971	a	1.0610	b	2.1695	a	0.2222	b	1.4252	a	1.1224	a	1.0473	a	0.3200	a	0.4323	a
PE_36	0.1618	b	0.3482	a	0.1173	b	0.3881	a	0.1738	b	0.3363	a	0.0894	b	0.3332	b	0.1863	b	0.3011	a	0.0500	b	0.2688	a	0.4523	a	0.5594	a	0.1598	b	0.6344	a	0.8896	b	1.2646	a	0.4723	b	1.3025	a	0.7694	b	1.4488	a	0.2905	b	1.0533	a	0.7349	a	0.7830	a	0.2323	a	0.4056	a
PG_34	0.2863	a	0.3472	a	0.2789	b	0.4676	a	0.3386	a	0.4541	a	0.2954	b	0.7915	b	0.4993	a	0.6133	a	0.3333	b	0.6305	a	0.3214	a	0.2901	a	0.2099	a	0.2861	a	1.0031	a	1.2126	a	0.5228	b	1.2552	a	0.8392	b	1.4246	a	0.3211	b	0.8327	a	0.6302	a	0.4654	a	0.2533	b	0.3851	a
PI_34	0.1212	b	0.2589	a	0.0463	b	0.2542	a	0.1292	b	0.2146	a	0.0486	b	0.2339	b	0.1647	b	0.3251	a	0.0129	b	0.2476	a	0.1363	b	0.2388	a	0.0154	b	0.1490	a	0.2511	b	0.3604	a	0.1053	b	0.2893	a	0.2096	b	0.3550	a	0.0545	b	0.2827	a	0.2796	b	0.3707	a	0.1006	b	0.2026	a
SQDG_34	2.1202	a	1.2953	b	2.4285	a	1.4403	b	2.1416	a	1.4351	a	2.1120	a	1.4000	b	2.2988	a	1.6146	b	2.4740	a	1.3576	b	1.8724	a	1.1112	b	2.1319	a	1.2299	b	1.4969	a	0.9251	b	1.7847	a	0.9928	b	1.7025	a	0.8827	b	2.4321	a	1.0891	b	1.0839	a	0.8188	b	1.7256	a	1.1263	b
SQDG_36	1.4513	a	1.0888	a	1.7041	a	1.4038	a	1.5798	a	1.5102	b	1.5247	a	1.7620	a	2.1438	a	1.8306	a	1.7439	a	1.5504	a	2.1151	a	1.4527	b	2.0272	a	1.5021	b	2.3732	a	2.1987	a	2.2388	a	2.4521	a	2.2513	a	2.0198	a	2.2181	a	2.0028	a	2.2257	a	1.7331	a	2.0217	a	1.9173	a

**Table 3:** Non-phospholipid and phospholipid species that were detected in the roots of seven cultivars under −P and +P treatments 5 and 10 days after transplanting (DAT). For each cultivar, different letters indicate significant (*p* < 0.05) differences between the −P and +P treatments.

Lipid Species	Koshihikari								Norin No.1								Akamai								Hiyadateine								Netaro								Akitakomachi								Kiyonishiki				−P			
	5 DAT				10 DAT				5 DAT				10 DAT				5 DAT				10 DAT				5 DAT				10 DAT				5 DAT				10 DAT				5 DAT				10 DAT				5 DAT				10 DAT			
	−P		+P		−P		+P		−P		+P		−P		+P		−P		+P		−P		+P		−P		+P						−P		+P		−P		+P		−P		+P		−P		+P		−P		+P		−P		+P	
DGDG_34	2.5461	a	0.6081	b	3.7276	a	1.1494	b	3.2980	a	0.6880	b	4.0855	a	0.9496	b	3.5617	a	0.9287	b	5.0312	a	0.8796	b	1.1670	a	0.3672	b	2.2844	a	0.6136	b	2.5635	a	0.7854	b	3.3035	a	0.7487	b	2.8166	a	0.6945	b	3.1561	a	0.8893	b	3.7333	a	1.9439	b	4.2200	a	1.2870	b
DGDG_36	0.7269	a	0.2053	b	1.3244	a	0.3358	b	0.9371	a	0.2777	b	1.5299	a	0.3806	b	1.1818	a	0.3322	b	2.1628	a	0.3855	b	0.2329	a	0.1072	b	0.7394	a	0.1702	b	0.8680	a	0.3745	b	1.1587	a	0.4197	b	0.9629	a	0.3560	b	1.2586	a	0.4309	b	1.6001	a	0.8916	b	1.4470	a	0.4171	b
GlcADG_343	0.4106	a	0.1195	b	0.6119	a	0.1458	b	0.5564	a	0.1271	b	0.6772	a	0.1357	b	0.5613	a	0.1433	b	0.8470	a	0.1890	b	0.3456	a	0.1315	b	0.6106	a	0.1574	b	0.6532	a	0.2023	b	0.8150	a	0.2485	b	0.7569	a	0.1769	b	0.8133	a	0.2466	b	0.6255	a	0.3071	b	0.8517	a	0.2211	b
GlcADG_36	0.0880	a	0.0204	b	0.1414	a	0.0272	b	0.1276	a	0.0216	b	0.1705	a	0.0268	b	0.1356	a	0.0276	b	0.2285	a	0.0386	b	0.0138	a	0.0076	a	0.0779	a	0.0111	b	0.1812	a	0.0250	b	0.1381	a	0.0303	b	0.1911	a	0.0181	b	0.1408	a	0.0249	b	0.3054	a	0.0667	b	0.3161	a	0.0360	b
MGDG_34	0.5489	a	0.1553	b	0.6651	a	0.4015	b	0.5732	a	0.2018	b	0.6701	a	0.2122	b	0.6598	a	0.2746	b	0.9403	a	0.1350	b	0.3639	a	0.1799	b	0.7719	a	0.3747	b	0.8789	a	0.4104	b	0.9220	a	0.2726	b	0.9975	a	0.2981	b	0.9070	a	0.3996	b	0.9165	a	0.4754	b	0.4695	a	0.1738	b
MGDG_36	0.3212	a	0.2969	a	0.5822	a	0.3555	b	0.3458	a	0.3945	a	0.6109	a	0.3572	b	0.4239	a	0.4713	a	0.9095	a	0.2774	b	0.0823	a	0.1240	b	0.3586	a	0.2272	b	0.5590	a	0.6593	a	0.7228	a	0.6979	a	0.6187	a	0.5673	a	0.6641	a	0.5624	a	1.1418	a	1.4052	a	0.4763	a	0.3417	a
PC_34	0.2117	b	0.4913	a	0.1854	b	0.6143	a	0.2462	b	0.3686	a	0.2788	b	1.2806	a	0.1719	b	0.6580	a	0.1315	b	1.1979	a	0.1368	b	0.2985	a	0.1323	a	0.3055	a	2.5237	b	3.3479	a	2.6537	a	3.4487	a	1.9142	b	2.7873	a	0.7333	b	1.1837	a	1.1934	a	1.6091	a	0.2022	b	0.5490	a
PC_36	0.1303	a	0.2518	a	0.1168	a	0.1324	a	0.1715	a	0.1984	a	0.1954	a	0.2847	a	0.0889	b	0.3044	a	0.0651	b	0.2963	a	0.0545	b	0.1299	a	0.0249	b	0.0227	a	0.5878	a	0.7231	a	0.6793	a	0.5593	a	0.4723	a	0.5195	a	0.1789	a	0.2214	a	0.7883	a	0.8056	a	0.0728	b	0.2472	a
PE_34	0.1163	b	0.3740	a	0.0791	b	0.2730	a	0.1263	b	0.2325	a	0.1197	b	0.6153	a	0.0985	b	0.4898	a	0.0434	b	0.4934	a	0.1027	b	0.3089	a	0.0683	a	0.2074	a	1.3741	b	2.2589	a	1.0417	b	2.4083	a	1.2522	b	2.1380	a	0.4045	b	1.0958	a	0.8298	a	1.1042	a	0.1353	b	0.4246	a
PE_36	0.0144	a	0.0313	a	0.0081	a	0.0167	a	0.0105	b	0.0257	a	0.0099	b	0.0499	a	0.0085	b	0.0632	a	0.0055	b	0.0570	a	0.0027	b	0.0159	a	0.0025	b	0.0021	a	0.1191	b	0.2708	a	0.1006	a	0.1557	a	0.0958	b	0.1888	a	0.0194	b	0.0528	a	0.2093	a	0.2604	a	0.0042	b	0.0662	a
PG_34	0.0012	b	0.0031	a	0.0011	a	0.0047	a	0.0014	b	0.0029	a	0.0010	b	0.0091	a	0.0015	b	0.0094	a	0.0016	a	0.0096	a	0.0018	a	0.0068	a	0.0014	b	0.0145	a	0.0486	a	0.0764	a	0.0287	b	0.1277	a	0.0402	b	0.0848	a	0.0088	b	0.0350	a	0.0313	a	0.0418	a	0.0024	b	0.0103	a
PI_34	0.0080	b	0.0250	a	0.0056	b	0.0359	a	0.0141	b	0.0286	a	0.0093	b	0.0550	a	0.0094	b	0.0487	a	0.0019	b	0.0824	a	0.0032	b	0.0120	a	0.0024	a	0.0069	a	0.0755	b	0.1454	a	0.0375	b	0.1668	a	0.0622	b	0.1268	a	0.0150	b	0.1060	a	0.0980	a	0.1744	a	0.0065	b	0.0319	a
SQDG_34	0.0345	a	0.0156	b	0.0577	a	0.1157	b	0.0434	a	0.0115	b	0.0584	a	0.0685	a	0.0483	a	0.0147	b	0.0741	a	0.0218	b	0.0225	a	0.0165	a	0.0450	a	0.0765	a	0.0651	a	0.0175	b	0.0927	a	0.0407	b	0.0681	a	0.0106	b	0.0833	a	0.0597	a	0.0708	a	0.0202	b	0.0791	a	0.0133	b
SQDG_36	0.0102	a	0.0031	b	0.0139	a	0.0041	b	0.0121	a	0.0044	b	0.0162	a	0.0061	b	0.0134	a	0.0067	b	0.0181	a	0.0053	b	0.0032	a	0.0014	b	0.0064	a	0.0024	a	0.0171	a	0.0068	b	0.0210	a	0.0055	b	0.0151	a	0.0058	b	0.0149	a	0.0062	b	0.0283	a	0.0095	b	0.0243	a	0.0054	b

## Data Availability

All data supporting the findings of this study are available within the paper and within its supplementary data published online.

## Supplementary Materials

The following supporting information can be downloaded at: https://www.mdpi.com/article/10.3390/plants12061365/s1, Figure S1: Low phosphorus tolerance of rice cultivars; Table S1: Shoot dry weight, P concentration, and P content of 42 rice cultivars grown in −P and +P levels. * indicates significant (*p* < 0.05) difference between −P and +P level; Table S2: Lipid species that were detected in the shoots of seven cultivars under −P and +P treatment 5 and 10 days after transplanting (DAT); Table S3: Lipid species that were detected in the roots of seven cultivars under −P and +P treatment 5 and 10 days after transplanting (DAT); Table S4: Correlation coefficient between log (−P/+P) of lipid species and low P tolerance values of seven cultivars.
