# Supplementary figures and images for: Lipidome Profiling of Phosphorus Deficiency-Tolerant Rice Cultivars Reveals Remodeling of Membrane Lipids as a Mechanism of Low P Tolerance
Source: Plants (Basel). 2023 Mar 18;12(6):1365. doi: 10.3390/plants12061365 (PMC10057753; doi:10.3390/plants12061365)

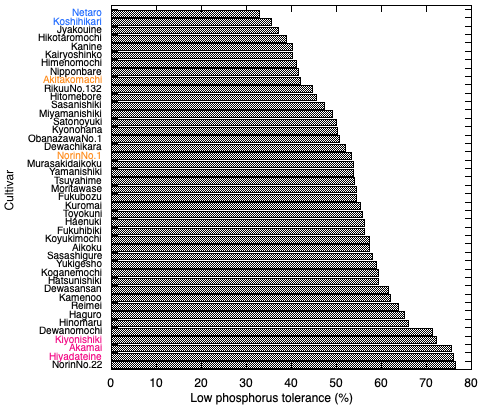

Supplement: Supplementary file 1 [file plants-12-01365-s001.zip › FigS1.tiff]
